# From CRISPR functional genomics to synthetic interventions: engineering antiviral strategies

**DOI:** 10.1128/jvi.00057-26

**Published:** 2026-03-30

**Authors:** Wenjie Qiao

**Affiliations:** 1 Institute of Infectious Diseases, Shenzhen Bay Laboratory 551667, Shenzhen, China; New York University Department of Microbiology, New York, New York, USA

**Keywords:** virus-host interactions, functional genomics, CRISPR screening, synthetic biology, antiviral strategies

## Abstract

Virus-host interactions govern infection outcomes and viral evolution, but host determinants that enable or restrict viral replication have been difficult to map comprehensively and in the right cellular contexts. Pooled CRISPR perturbation screens now provide scalable, mechanistic entry points into host dependency and restriction landscapes across diverse viruses. Recent extensions, including single-cell readouts, imaging and spatial phenotyping, organoid models, and *in vivo* selection, are shifting the field from static hit lists toward contextual maps that explain how host pathways and cell states shape permissiveness. In parallel, synthetic biology is translating these maps into programmable intervention classes, including receptor decoys and binders that intercept entry, conditional protein depletion systems that modulate host factors with temporal control, gene circuits that couple infection sensing to tailored responses, and engineered immune cells with tunable antiviral functions. This review highlights conceptual and technical advances that connect CRISPR functional genomics to synthetic antiviral design, summarizes emerging principles that generalize across viral families, and discusses constraints that will determine whether screen-nominated mechanisms can be engineered into effective and safe antiviral strategies.

## INTRODUCTION

Viruses are obligate intracellular parasites that rely on host cellular machinery to complete their life cycles. From attachment and entry to replication, assembly, and egress, viral infection is shaped by specific host factors and pathways. Understanding these virus-host interactions is therefore central to virology, as it reveals how viruses exploit cellular systems and how hosts deploy intrinsic and innate defenses, forming the conceptual foundation for antiviral development (1–3).

**Fig 1:**
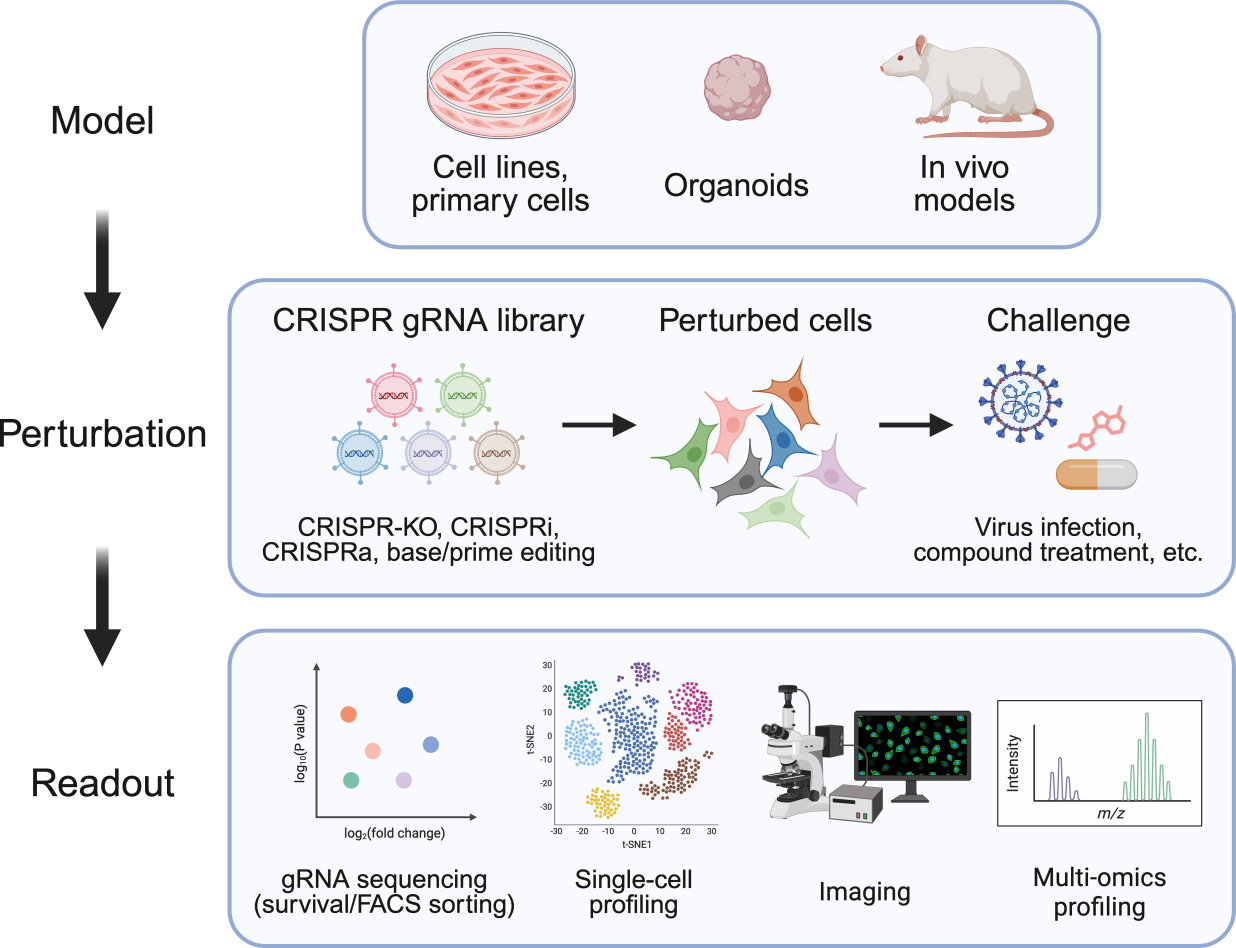
The CRISPR functional genomics toolkit for virology. CRISPR perturbation libraries (KO, CRISPRi, CRISPRa, and base/prime editing) are applied to diverse model systems, including cell lines/primary cells, organoids, and *in vivo* models, followed by viral challenge or other selective pressures. Phenotypes are quantified using sequencing-based guide enrichment, single-cell profiling, imaging, and multi-omics readouts to identify host factors and pathways that modulate infection and antiviral responses.

Over the past decade, advances in genome engineering and systems-level biology have reshaped how these interactions are mapped and manipulated. The advent of clustered regularly interspaced short palindromic repeats (CRISPR) technologies introduced a programmable, scalable, and versatile toolkit for perturbing gene function and regulation, enabling pooled loss- and gain-of-function genetics in mammalian cells (4–6). CRISPR-based functional genomics encompasses multiple perturbation modalities, including CRISPR knockout (CRISPR-KO), interference (CRISPRi), activation (CRISPRa), base editing, and prime editing, allowing complementary views of host dependence—from hypomorphic regulation and pathway tuning to precise sequence changes (7–10). These tools have been deployed extensively in transformed cell models and, increasingly, in more physiological systems including primary cells, organoids, and select *in vivo* models. Where feasible, they can also be paired with higher-content readouts such as single-cell and multi-omic profiling (11–15) (Fig. 1). Applying these approaches in virology has generated increasingly contextual maps of host dependency factors, antiviral restriction pathways, and regulatory nodes that shape viral tropism and pathogenesis (16–18). The field is moving beyond cataloging gene-level requirements toward defining network architectures and cellular states that determine susceptibility or resistance to infection.

Parallel advances in synthetic biology provide a design-oriented framework for translating mechanistic insights into programmable antiviral strategies. By enabling modular engineering of biomolecules and gene circuits, synthetic biology makes it possible to couple infection-linked sensing to user-defined cellular outputs, such as antiviral effector expression, conditional apoptosis, immune signaling, or programmable secretion, thereby shifting intervention logic from inhibiting viral enzymes to engineering host responses (19–22). This design space spans multiple intervention classes: receptor decoys or binders that intercept entry, conditional protein depletion or degradation systems, state reprogramming approaches, engineered immune cells, and integrated delivery and safety architectures.

These fields are converging toward an emerging programmable virology paradigm, in which functional genomics maps actionable virus-host mechanisms and synthetic biology implements them as controllable interventions. This review synthesizes that convergence by highlighting (i) recent advances in CRISPR functional genomics for viral systems, (ii) biological insights into host dependency and restriction architectures, and (iii) translational opportunities and constraints for converting these insights into engineered antiviral strategies.

## CRISPR FUNCTIONAL GENOMICS AT SCALE: MAPPING THE VIRUS-HOST INTERFACE

CRISPR-based functional genomics has transformed the study of virus-host interactions by enabling systematic perturbation at genome scale with programmable tools and standardized screening workflows. Earlier RNA interference approaches generated important early host-factor maps but were often constrained by variable knockdown efficiency across reagents and off-target effects (23, 24). In contrast, CRISPR platforms support robust loss- and gain-of-function screening, can be applied to both coding and non-coding elements, and increasingly enable more precise, variant- and allele-resolved questions (25, 26). Collectively, these capabilities have accelerated efforts to chart host dependency and restriction landscapes across many viral systems and nominate mechanistic vulnerabilities for therapeutic design (16, 18).

### A toolkit of CRISPR functional genomic approaches

A major strength of CRISPR functional genomics is pooled screening, in which large perturbation libraries are introduced in parallel and selected using infection-linked phenotypes (survival, reporter activity, antigen positivity, or quantitative viral RNA/protein readouts), with guide abundance recovered by sequencing. The foundational modality is CRISPR-KO, where CRISPR-associated protein 9 (Cas9) nuclease introduces indels disrupting gene function (4). KO screens are well suited for identifying strong, non-redundant host dependencies and have repeatedly uncovered core cellular machinery co-opted by viruses, such as endoplasmic reticulum (ER)-associated complexes required for flavivirus replication (27).

However, complete gene ablation is not always feasible. Many viral phenotypes rely on essential host functions, where knockout triggers lethality or compensatory stress responses that obscure infection-specific effects. In such settings, CRISPRi provides a complementary strategy: catalytically inactive Cas9 (dCas9) fused to transcriptional repressors (e.g., KRAB) achieves tunable, reversible transcriptional knockdown without altering DNA sequence (28, 29). This graded repression is particularly useful for probing dose-sensitive dependencies and essential pathways. For example, a targeted CRISPRi screen for severe acute respiratory syndrome coronavirus 2 (SARS-CoV-2) spike binding identified the bromodomain protein BRD2 as a regulator of ACE2 expression and SARS-CoV-2 infection (30). Conversely, CRISPRa expands screening into gain-of-function space by coupling dCas9 to transcriptional activators (e.g., VP64, p65, VPR), upregulating endogenous gene expression to discover restriction factors or antiviral programs weakly expressed basally (31, 32). For example, CRISPRa screening has identified factors restricting Zika virus infection, including interferon (IFN)-linked effectors such as IFN-λ2 and IFI6 (33).

More recently, base editing and prime editing have moved functional genomics toward nucleotide-resolution interrogation. Base editors enable targeted point mutations without double-strand breaks; prime editors support a wider range of substitutions and small indels (8, 26, 34, 35). These approaches functionally annotate host variants and map structure-function relationships in dependency factors. For instance, saturation prime editing of NPC1 demonstrated high-throughput endogenous mutational scanning for a key filovirus host factor (9).

### Advanced screening paradigms: resolution and context

Early viral CRISPR screens predominantly used transformed cell lines with relatively low-dimensional readouts. Recent innovations have expanded both resolution and physiological relevance.

Integrating CRISPR perturbations with single-cell transcriptomics (e.g., Perturb-seq, pooled CRISPR screens with single-cell RNA-seq readout; CROP-seq, CRISPR droplet sequencing) enables simultaneous recovery of perturbation identity and cell state, generating perturbation-response maps that capture direct and downstream consequences of host-factor disruption (14, 36, 37). Perturb-seq has revealed that seemingly uniform cell populations exhibit divergent infection trajectories: in human cytomegalovirus infection, single-cell resolution distinguished cells supporting productive replication from those mounting abortive responses, with distinct transcriptional signatures that bulk measurements average away. Similar heterogeneity in cellular permissiveness and antiviral responses has been documented in SARS-CoV-2 systems (38, 39).

In parallel, imaging-based CRISPR screens extend pooled genetics to complex phenotypes: organelle remodeling, protein localization, and subcellular signaling (40). Phenotype-positive cells can be isolated via microraft-based approaches or enriched by fluorescence-activated cell sorting using photoactivatable fluorescent proteins, with perturbations recovered by sequencing (41–43). Barcode readout via *in situ* sequencing preserves spatial context (44). These modalities are promising for infection phenotypes not well captured by bulk survival or simple reporters. Using this strategy, optical pooled and imaging-based CRISPR screens have identified host regulators of Sendai virus and Ebola virus infection (45, 46).

Moving toward more complex model systems represents another significant advance. Organoid platforms better recapitulate tissue architecture, cellular heterogeneity, and differentiation states than traditional cell lines, potentially revealing host dependencies undetectable in simpler two-dimensional systems. Organoid infection models increasingly support virology questions about tropism, infection dynamics, and cell-type-specific responses and are established for SARS-CoV-2 and other clinically important viruses (47–49). Pooled CRISPR screening has been adapted for organoids across multiple biomedical contexts, including cancer, neurological disease models, and dissecting gene-drug interactions (50–54), yet direct combination with productive viral infection remains limited. A CRISPR-engineered intestinal organoid biobank used to test coronavirus entry requirements confirmed ACE2 and TMPRSS2 as key entry determinants (55), highlighting both opportunity and practical barriers for scalable physiological viral screening.

*In vivo* CRISPR screening extends discovery into intact tissues and immune environments, where gene function can differ substantially from *in vitro* settings (13, 56). While *in vivo* pooled perturbation is maturing in other fields, screening specifically for viral infection phenotypes remains underexplored. Proof-of-principle work nonetheless supports feasibility: lipid-nanoparticle delivery of single-guide RNAs targeting Ebola entry factor NPC1 in Cas9 mice protected animals against surrogate viral challenge, demonstrating feasibility of interrogating host dependencies directly in living organisms (57).

### Interpreting CRISPR screens: from hit identification to mechanistic insight

As CRISPR screening data sets grow, the bottleneck shifts from hit calling to biological interpretation: converting enriched or depleted genes into mechanistic models that generalize across conditions and guide translation. Several methodological directions help close this gap.

First, combinatorial and multiplex perturbations address genetic redundancy and pathway buffering, which can otherwise yield false negatives. Dual- and multi-gene knockout approaches, including multiplex CRISPR-associated protein 12a (Cas12a) platforms, enable systematic mapping of synthetic lethal, masking, and buffering interactions among paralogous or functionally related genes (58–61). For virology, these designs are relevant where viruses reroute host pathways or multiple host factors provide overlapping support.

Second, multi-omic integration improves prioritization and mechanistic interpretation. Cross-referencing CRISPR hits with proteomics (62, 63), metabolomics (64, 65), glycomics (66, 67), and transcriptomic/spatial data sets (39, 68) helps place genes into functional modules rather than treating them as isolated entries. Integrating genetic dependencies with viral-host protein interaction maps can nominate direct mechanistic links between viral proteins and essential host machinery (69, 70). RNA-centric proteomics (e.g., ChIRP-MS, comprehensive identification of RNA-binding proteins by mass spectrometry) identifies host proteins physically engaging viral RNAs that can be cross-validated genetically (62, 71). Network-based clustering reveals functional modules, such as vesicle trafficking, lipid metabolism, or innate immune signaling, that operate as coordinated units during infection. Such cross-validated nodes represent central hubs of viral dependency and provide key mechanistic anchors.

Third, interpreting CRISPR screening hits requires explicit attention to cellular context. Viral dependencies can differ across cell types and culture systems due to receptor expression, innate immune tone, differentiation state, and metabolic configuration. For SARS-CoV-2, recurrent modules such as endosomal trafficking and cholesterol homeostasis emerge across studies, yet specific hits and effect sizes vary with cellular model (72, 73). This argues for validation across multiple contexts to separate broadly acting dependencies from model-specific ones.

Together, these advances shift CRISPR screening from hit lists to mechanistic maps, resolving pathway structure, interaction logic, and context dependence. CRISPR hits are not endpoints but starting points: they motivate targeted validation, define coherent host modules exploited or opposed during infection, and sharpen hypotheses about where intervention is most likely effective.

## BIOLOGICAL INSIGHTS FROM GENETIC CRISPR SCREENING

CRISPR screens have become central for dissecting virus-host interactions, enabling unbiased identification of host factors governing infection across diverse viral families. Beyond hit lists, convergent screens increasingly reveal recurring dependency modules, spanning entry determinants, endolysosomal activation environments, ER proteostasis, and lipid remodeling, while clarifying how cell state and context reshape genetic requirements (18). Table 1 organizes representative screens by mechanistic module discussed in this section, illustrating how the field is moving from virus-centric catalogs toward pathway-level understanding.

**TABLE 1:** Representative viral CRISPR screens discussed, organized by mechanistic module

Virus	Screen type	Key finding(s)	Mechanistic module
Coronaviruses	CRISPR-KO CRISPRa	ACE2 as dominant receptor; TMPRSS2-driven plasma membrane entry vs cathepsin-dependent endolysosomal entry (72–74)	Entry receptors/cofactors
Venezuelan equine encephalitis virus	CRISPR-KO	LDLRAD3 identified as entry receptor (75)	Entry receptors/cofactors
Alphaviruses	CRISPR-KO	LDLR, VLDLR, and LRP8/ApoER2 mediate entry across alphaviruses (76, 77)	Entry receptors/cofactors
Severe fever with thrombocytopenia syndrome virus	CRISPR-KO	LRP1 identified as entry factor (78)	Entry receptors/cofactors
Yellow fever virus	CRISPR-KO	LRP1, LRP4, and VLDLR implicated in entry (79)	Entry receptors/cofactors
Tick-borne encephalitis virus	CRISPR-KO	LRP8/ApoER2 reported as an entry receptor (80)	Entry receptors/cofactors
Enterovirus D68	CRISPR-KO	MFSD6 identified as entry receptor (81)	Entry receptors/cofactors
Human parechovirus	CRISPR-KO	MYADM identified as a post-entry receptor (82)	Entry receptors/cofactors
Ebola virus	CRISPR-KO	GNPTAB (M6P tagging) required for lysosomal hydrolase/cathepsin-dependent entry (83)	Endolysosomal protease supply
Cathepsin-dependent viruses	CRISPR-KO	LYSET/TMEM251 essential for lysosomal enzyme transport and protease supply (63)	Endolysosomal protease supply
Flaviviruses	CRISPR-KO	OST complex and ER biogenesis modules required for infection (27)	ER proteostasis
Flaviviruses	CRISPR-KO + follow-up	EMC required for NS4A/NS4B topology and replication organelle formation (27, 84, 85)	ER proteostasis
Dengue and Zika viruses	ChIRP-MS + CRISPR-KO	RRBP1 and vigilin identified as proviral RNA-binding proteins (62)	ER proteostasis
Yellow fever virus	CRISPR-KO	Ribosome biogenesis factors (SBDS, SPATA5) required for infection (86)	ER proteostasis
Coronaviruses	CRISPR-KO + follow-up	SMARCA4 and DYRK1A regulate ACE2 expression and susceptibility (74, 87)	ER proteostasis
Sindbis virus	CRISPR-KO	COG4 implicated in infection (88)	ER proteostasis
Influenza A virus	CRISPR-KO	COG complex integrity influences entry/fusion and viral spread (89)	ER proteostasis
Flaviviruses/β-coronaviruses	CRISPR-KO	TMEM41B and VMP1 required for replication organelle biogenesis (90, 91)	Lipid remodeling pathways
SARS-CoV-2	CRISPR-KO	NPC1/NPC2 cholesterol trafficking modulates infection in certain contexts (72)	Lipid remodeling pathways
Human immunodeficiency virus	Virus-packageable CRISPR-KO	MxB, IFITM1, BST2, and TRIM5α identified as dominant ISG effectors (92)	Innate immune signaling
Flaviviruses	CRISPR-KO	IFI6 blocks replication organelle formation (93)	Innate immune signaling
Zika virus	CRISPRa	IFN-λ2 and IFI6 identified as restriction factors (33)	Innate immune signaling
Zika virus	CRISPR-KO	AMOTL2 tunes STAT1 signaling amplitude and antiviral outcomes (94)	Innate immune signaling

### Entry receptors and cofactors

CRISPR screening has been especially impactful at the virus-host interface because entry is often the most discrete and genetically tractable bottleneck. For SARS-CoV-2, independent genome-wide screens converged on ACE2 as the dominant receptor while repeatedly recovering factors partitioning entry routes across cell types. Screens highlight TMPRSS2-driven plasma membrane entry versus endolysosomal, protease-dependent routes relying on cathepsins—differences that become especially apparent in TMPRSS2-low contexts, where cathepsin-linked dependencies become prominent (72–74). This route plasticity framing helps reconcile why screens share core dependencies yet diverge in secondary hits depending on cellular model and infection conditions.

Beyond coronaviruses, genetic receptor discovery increasingly reveals viruses converging on receptor families combining broad expression with endocytic competence. The LDL receptor family appears repeatedly across distinct viral lineages. LDLRAD3 was identified as a receptor for Venezuelan equine encephalitis virus (75), while LDLR, VLDLR, and LRP8/ApoER2 mediate entry for multiple alphaviruses (76, 77). LRP1 serves as an entry factor for severe fever with thrombocytopenia syndrome virus (78), and recent work implicates LRP1, LRP4, and VLDLR in yellow fever virus entry (79). Additionally, LRP8 has been identified as an entry receptor for tick-borne encephalitis virus (80), extending LDLR family involvement across flaviviruses. These findings suggest entry genetics expose reusable receptor scaffolds that viruses repeatedly exploit, useful for mechanistic comparison and for conceptualizing broader entry-blocking strategies.

CRISPR-enabled receptor discovery has also broadened the entry landscape beyond canonical immunoglobulin-like or lectin families. Genome-scale approaches identified the multipass membrane transporter MFSD6 as an enterovirus D68 receptor and MYADM as an essential receptor for human parechoviruses (81, 82). These discoveries underscore a recurring lesson: physiologically important receptors need not belong to stereotypical classes, and unbiased genetics remains one of the most effective ways to surface unexpected but actionable entry determinants.

### Membrane trafficking and metabolism

CRISPR screens have repeatedly underscored that many viruses depend on organelle logistics, particularly trafficking pathways tuning protease availability, glycoprotein processing, and membrane composition. The mannose-6-phosphate (M6P) lysosomal enzyme trafficking axis governs delivery of cathepsins and other hydrolases required for endosomal/lysosomal activation. In Ebola virus infection, a genome-wide CRISPR screen identified GNPTAB (encoding subunits of the GlcNAc-1-phosphotransferase required for M6P tagging) as a strong proviral dependency, consistent with a requirement for properly equipped lysosomes during entry (83). More broadly, CRISPR screens using cathepsin-dependent viruses as functional probes identified LYSET/TMEM251 as an essential regulator of lysosomal enzyme transport, mechanistically linking disrupted M6P tagging and lysosomal hydrolase mislocalization to loss of cathepsin-dependent entry (63).

A second recurring trafficking theme involves the Golgi and post-Golgi secretory system, supporting glycoprotein maturation, membrane flow, and the organization of glycosylation enzymes. Components of the conserved oligomeric Golgi (COG) complex have emerged as proviral factors in multiple viral systems. For example, COG4 was implicated in a CRISPR-Cas9 screen with Sindbis virus, and orthopoxvirus studies show COG complex integrity influences entry/fusion and viral spread (88, 89).

Beyond trafficking, membrane remodeling and lipid homeostasis emerge as conserved dependency modules. ER-resident lipid scramblases TMEM41B and VMP1 recur across independent screens as required for flavivirus infection and β-coronavirus replication organelle biogenesis, consistent with roles in ER membrane remodeling and lipid mobilization needed to form viral RNA replication organelles (90, 91, 95). Genes controlling cholesterol trafficking influence infection through at least two distinct mechanisms. In SARS-CoV-2 CRISPR screens, cholesterol transporters NPC1/NPC2 modulate infection in certain cellular contexts, consistent with effects on endosomal membrane properties (72). In filoviruses, NPC1 serves as an essential intracellular entry receptor by directly engaging cleaved viral glycoprotein, a function separable from its canonical role in cholesterol export, illustrating how the same host factor can be hit by screens for fundamentally different reasons across viral systems (83).

### Protein-processing and gene-regulatory pathways

CRISPR screens have clarified that the limiting steps for many viruses extend beyond receptor engagement or membrane remodeling to the host’s capacity to produce, process, and correctly localize viral proteins at the ER. In flaviviruses, genome-scale CRISPR knockout studies converge on ER-resident machineries governing co-translational translocation, signal peptide handling, and N-linked glycosylation, including strong dependence on the oligosaccharyltransferase complex and other ER biogenesis modules (27). These genetic findings align with flaviviral polyprotein expression requiring coordinated insertion and processing at the ER to generate functional replication and assembly components.

ER quality control represents a second dependency layer highlighted by screens, pathways ensuring correct folding/topology of viral membrane proteins and managing misfolded intermediates generated under high viral protein load. Mechanistic follow-up showed the ER membrane protein complex (EMC) is required for correct topology and stable expression of flaviviral multi-pass nonstructural proteins (notably NS4A/NS4B), linking genetic hits to a concrete biogenesis bottleneck for replication complex formation (84, 85). More broadly, ER proteostasis and degradation pathways recur in host-factor maps for flaviviruses, consistent with viruses exploiting ER folding and turnover capacity to maintain productive infection (27).

CRISPR and orthogonal functional genomics also point to post-transcriptional control nodes coupling viral RNAs to productive translation and genome replication. An RNA-centric approach integrated with genome-scale knockout screening identified ER-associated RNA-binding proteins such as RRBP1 and vigilin as functional proviral factors in dengue and Zika virus infection, supporting a model in which viral RNAs engage specialized host ribonucleoprotein environments promoting efficient RNA utilization (62). Consistent with this, RACK1 recruits vigilin and SERBP1 to promote dengue virus replication, linking viral RNA to the translation machinery (96). Screens have further highlighted that ribosome supply and maturation can be limiting: a genome-scale CRISPR screen with yellow fever virus recovered ribosome biogenesis factors including SBDS and SPATA5/AFG2A, connecting rRNA processing/ribosome assembly capacity to viral protein synthesis output (86).

Finally, CRISPR screens in coronaviruses illustrate how host requirements can operate “upstream” of protein processing by shaping gene-regulatory states that gate susceptibility, particularly receptor availability. In a multi-coronavirus CRISPR screening framework, chromatin-associated regulators including SMARCA4 were implicated as proviral in part through control of ACE2 expression programs (74). Complementary mechanistic work supports DYRK1A promoting transcriptional accessibility at receptor loci (including ACE2 and DPP4), reinforcing that epigenetic/transcriptional control of entry determinants can be a major source of model- and lineage-dependent variability in host-factor maps (74, 87).

### Innate immunity and restriction factors

CRISPR-based functional genomics has sharpened the study of cell-intrinsic antiviral defense by separating three often-conflated layers: upstream signaling components, effector restriction factors, and virus- (or strain-) specific escape liabilities. In human immunodeficiency virus (HIV), virus-packageable CRISPR knockout screens revealed that IFN-mediated inhibition is largely dictated by combined activity of a small panel of interferon-stimulated genes (ISGs), including MxB, IFITM1, Tetherin/BST2, and TRIM5α. A follow-up screen using a distinct HIV-1 isolate recovered an overlapping but non-identical set of dominant effectors, emphasizing that restriction landscapes can be strain-conditioned and shaped by viral genotype as well as host state (92, 97).

In flaviviruses, CRISPR screens have been instrumental in dissecting the IFN-mediated restriction machinery. Loss-of-function screens repeatedly recover canonical signaling components (e.g., IFNAR1, JAK1, STAT2, IRF9) as essential for establishing antiviral states. More revealing are effectors mapping IFN activity onto specific viral cell biology. For example, CRISPR screening identified IFI6 as an IFN-inducible, ER-resident restriction factor that blocks flavivirus replication by preventing virus-induced ER membrane invaginations, providing a direct mechanistic bridge between innate signaling and replication-organelle biogenesis (93). Complementary gain-of-function CRISPRa screening in Zika virus highlighted protective programs weakly expressed basally that become impactful upon induction (33). More recently, a Zika-focused CRISPR knockout screen identified AMOTL2 as an antiviral factor tuning type I IFN responses by modulating STAT1 abundance and activation dynamics, illustrating that restriction can also be controlled by regulators shaping signaling amplitude rather than acting as direct viral antagonists (94).

A broader insight from CRISPR-enabled ISG interrogation is that ISGs are not uniformly restrictive; some act as context-dependent modulators with virus-specific directionality. LY6E, a glycophosphatidylinositol-anchored IFN-inducible surface protein, exemplifies this duality: it restricts multiple coronaviruses by impairing spike-mediated membrane fusion (98), yet enhances infection by select endocytosis-dependent viruses, including influenza A virus and yellow fever virus, by promoting post-endosomal uncoating (97, 99).

Collectively, CRISPR-based functional genomics has reframed innate immunity in genetic terms: IFN phenotypes can often be reduced to a limited set of dominant effectors, shaped by viral genotype and cellular context, and linked to discrete mechanistic choke points. This restriction layer complements dependency modules, together providing a principled map of where viruses are most constrained by host biology.

## TRANSLATIONAL SYNTHETIC DESIGN: FROM GENOMIC INSIGHT TO ENGINEERED INTERVENTION

**Fig 2:**
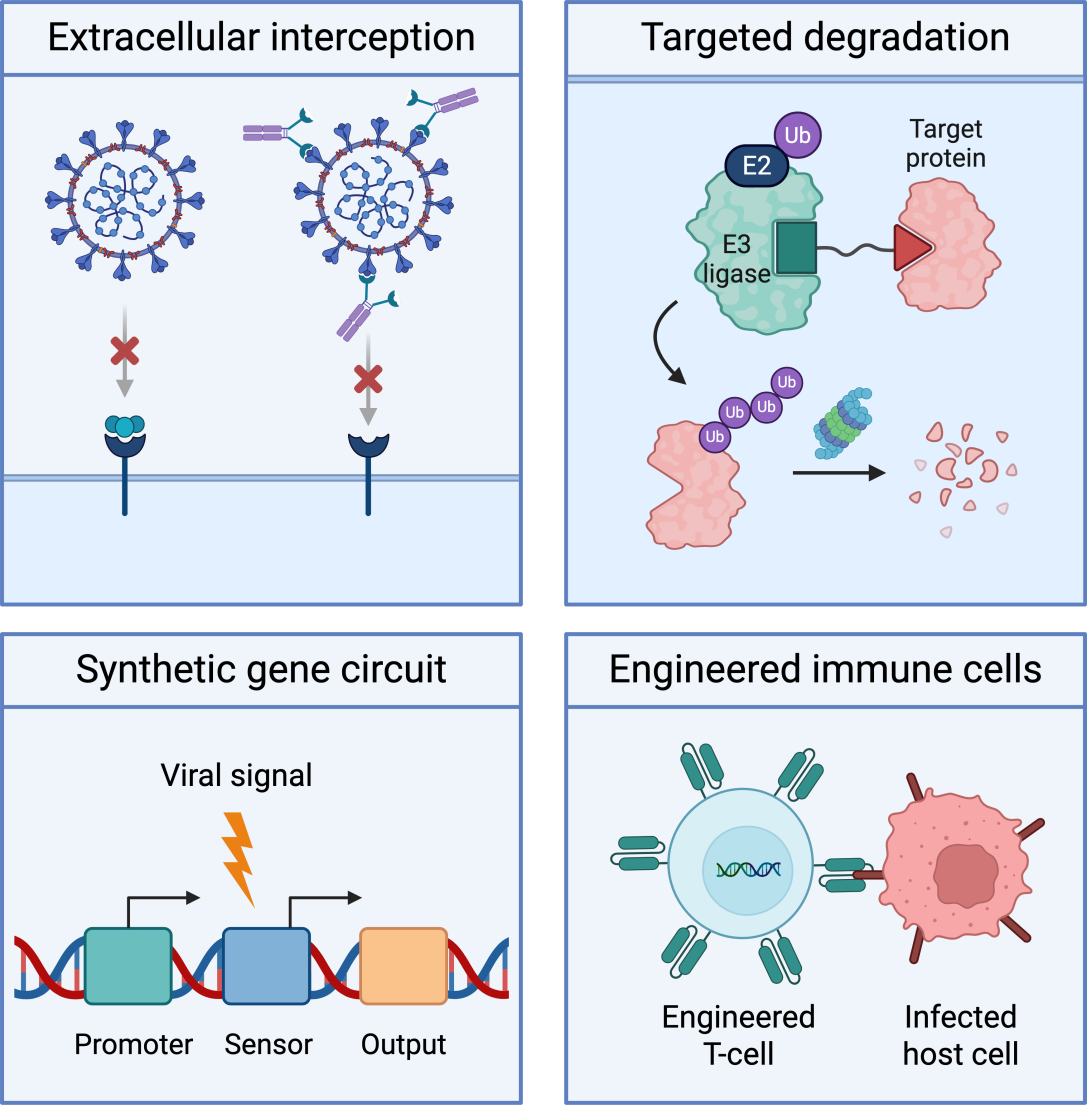
Translating virus-host insights into synthetic antiviral intervention classes. Four design patterns illustrate translation of mechanistic insights into engineered strategies: extracellular interception of entry through receptor-blocking binders and viral traps; targeted protein degradation to modulate key factors; infection-sensing gene circuits coupling viral signals to programmed outputs; and engineered immune cells recognizing and eliminating infected host cells.

A key promise of CRISPR functional genomics in virology extends beyond discovery to operationalizing these mechanisms into programmable intervention classes (16, 100). As the field moves from gene-centric hit lists toward contextual and state-resolved maps of permissiveness, these data sets become design inputs specifying what to sense or modulate, when to act during infection, and where to act across relevant cell types and tissues. Synthetic biology provides a modular toolkit to implement these specifications, from extracellular interception of viral entry to intracellular conditional control and engineered immune functions (19, 20). Together, these approaches reframe antivirals as controllable, context-specific host interventions rather than constitutive inhibitors of viral enzymes (Fig. 2).

### Converting screen hits into intervention hypotheses: a design workflow

A practical translation workflow begins by re-annotating CRISPR screen hits into mechanistic modules, such as entry receptors/cofactors, endolysosomal protease supply, ER proteostasis, lipid remodeling pathways, or innate immune signaling gates, rather than treating them as independent genes. This module-first view matters because synthetic interventions often act at the function level, and functional modules tend to generalize across viral families even when specific genes do not (19, 20).

Context dependence, increasingly evident across CRISPR screens performed in different cell types or differentiation states, becomes informative (101, 102). Differences in entry routes, receptor expression, metabolic configuration, and baseline innate immune tone help define where a given intervention is likely effective and where toxicity may arise. In this framing, CRISPR maps define actionable control points, while engineering decisions determine feasibility, including delivery strategy, reversibility, tunability, specificity, and safety.

### Entry interception and receptor-level strategies

Viral entry is often the most discrete bottleneck and therefore one of the most engineerable stages (103). Where CRISPR screens converge on dominant receptors or receptor families, one translation route is engineering extracellular molecular interceptors that block infection before intracellular replication programs engage. Structure-guided protein engineering has enabled the design of receptor decoys recapitulating viral binding interfaces while eliminating downstream signaling. Computationally engineered ACE2 decoys with enhanced affinity and resistance to viral escape provide a clear example (104). *De novo* designed miniprotein binders targeting viral receptor-binding domains demonstrate how compact and hyperstable scaffolds can achieve extremely high affinity and potent antiviral activity, illustrating the synthetic principle of constructing minimal functional binding parts (105, 106).

CRISPR-derived context information is essential for determining when these approaches are likely to succeed. Screens distinguish receptor-limited entry, where interception strategies are effective, from cases where downstream steps dominate and entry blockade offers limited benefit (72, 107). This distinction guides choice between extracellular interception and alternative host-directed strategies. Related approaches include surface rewiring, where regulated expression or sequestration of receptors and cofactors reduces productive entry in vulnerable cellular states. Compared with small-molecule inhibitors, these strategies can be cell-type restricted and designed to be inducible or reversible, reducing concerns about chronic perturbation of essential host functions. Engineered biologics such as host-targeting antibodies offer complementary extracellular means of receptor blockade, steric masking, or induced internalization when genetic modulation is impractical.

### Conditional control of host dependency factors: timed modulation and protein depletion

Many proviral host dependencies are essential cellular functions, making constitutive inhibition untenable. Synthetic biology enables conditional control, allowing interventions to act only within defined temporal windows or cellular states (29, 108, 109). One generalizable translation is dose-controlled modulation using programmable nucleic acid-based modalities such as CRISPRi or inducible epigenetic editors (29, 110). These systems support graded repression rather than complete loss of function and can be deployed transiently or under external control. Context-aware CRISPR screens revealing dose-sensitive dependencies are particularly valuable because they define therapeutic windows where viral replication is impaired while cellular viability is preserved (111). Complementary RNA-targeting approaches, including small interfering RNA and antisense oligonucleotides, provide reversible transcript-level modulation without permanent genomic alteration (112).

Additional control can be imposed at the protein level through engineered degradation. Synthetic degrader systems enable transient depletion of host factors required only during narrow infection stages, offering strong temporal precision and rapid reversibility (108, 109). Proof-of-principle studies demonstrate host-directed degradation can suppress viral replication by targeting dependencies involved in translation, chaperone function, or protein trafficking (113, 114). As degrader technologies expand to include engineered proteins and computationally designed binders, protein degradation emerges as a synthetic control axis for navigating host dependency landscapes defined by CRISPR screens (115, 116).

### Infection-sensing gene circuits and engineered cellular states

CRISPR functional genomics increasingly indicates that viral permissiveness is a property of cellular state as much as gene identity. It is shaped by differentiation status, metabolic configuration, and baseline innate immune tone. This is reflected in the strong context dependence across screens, where both identity and effect size of host factors vary with cellular background (101, 102). Synthetic biology can capitalize on this insight by reprogramming host cells into states intrinsically less supportive of infection, ideally in a conditional and tissue-restricted manner.

Engineered gene circuits provide a route to impose preemptive or inducible antiviral states using modular sensing and actuation (117). Circuits informed by CRISPRa screens of restriction factors could selectively amplify antiviral programs while avoiding full interferon activation, decoupling antiviral efficacy from inflammatory toxicity (118). Infection-linked sensing further improves specificity. Protease-responsive modules exploit the fact that viral protease activity arises only during productive infection and can be converted into programmable transcriptional or cell-fate outputs (119). RNA-responsive regulators provide an additional sensing channel by detecting defined viral RNA signatures (120). Together, these approaches represent a shift from targeting individual host factors to engineering cellular states, treating infection as a systems-level perturbation that can be countered by reprogramming host cell behavior. Crucially, reprogramming cellular states must be balanced against risks of disrupting normal physiology, as persistent activation of antiviral programs could impair essential cellular functions, trigger inflammatory pathology, or disturb tissue-level homeostasis. These concerns argue for strategies that are transient, tissue-restricted, or coupled to infection sensing rather than constitutive.

### Engineered immune functions, delivery integration, and safety

Synthetic immune engineering extends translational design beyond intrinsic cellular responses to immune cells as programmable agents. Here, CRISPR functional genomics contributes not only by enabling efficient multigene editing, but also by nominating immune-intrinsic regulators of activation, trafficking, and antiviral effector programs that can be engineered into cells (11, 13). In this framework, antiviral immunity is not only amplified but actively shaped through engineered control of recognition thresholds, activation timing, trafficking, and effector composition (121, 122). Advances in synthetic receptor and gene circuit platforms enable customized sensing and response programs, while CRISPR-based genome engineering supports efficient multigene editing directly in primary immune cells (123, 124). Synthetic receptor architectures demonstrate how extracellular recognition couples to programmable transcriptional outputs, enabling combinatorial logic that improves specificity when no single marker uniquely defines infected states (21, 125).

Although most established in oncology, antiviral applications of engineered immune cells provide compelling proof of principle across viral systems, including HIV and hepatitis B virus (126, 127). Genome engineering strategies enabling large knock-ins at defined loci further support integration of synthetic receptors, control circuits, and regulatory modules without viral vectors (128, 129). Because potent immune effectors can damage healthy tissue, synthetic immune engineering is tightly coupled to safety considerations. Inducible cell ablation mechanisms provide clinically validated safeguards that allow engineered cells to be eliminated in the event of severe toxicity (130).

Across all engineered antiviral strategies, delivery and system-level integration remain decisive constraints. Functional genomics has revealed that delivery efficiency, tissue tropism, and expression durability are governed by host genetic determinants, directly linking CRISPR-derived maps to delivery design (131, 132). Studies identifying host factors required for adeno-associated virus entry and transduction demonstrate vector performance reflects host gene networks rather than capsid properties alone, enabling host-centric approaches to program tissue tropism genetically (133). Different delivery modalities impose distinct constraints. Viral vectors support durable expression but raise concerns about immunity and host variability; lipid nanoparticles enable transient, repeatable delivery suited to time-limited interventions (134, 135). For adoptive cell therapies, *ex vivo* genome engineering allows complex systems to be assembled and validated prior to clinical deployment. Ultimately, engineered antiviral feasibility hinges on balancing mechanistic validity across biological contexts, delivery to the appropriate cells at the appropriate times, and safety with reversibility when host pathways are manipulated.

## CHALLENGES AND FUTURE DIRECTIONS

The integration of CRISPR functional genomics with synthetic biology is enabling a new discover-design-build cycle for virology. In practice, the main bottlenecks lie at the interfaces between screening and biology, where the meaning of a hit depends on context, and between design and deployment, where feasibility is constrained by safety, targeting, and *in vivo* performance (18, 20). Closing these gaps is essential for turning CRISPR-derived maps into actionable design inputs rather than descriptive catalogs.

On the discovery side, CRISPR screens are powerful because they are scalable and relatively unbiased, but they are also conditional experiments. Dependencies shift with model choice, infection conditions, and readout design (73). The field is moving toward mechanistic portability, identifying modules and causal logic that persists across models while explicitly flagging context-locked dependencies (18). This shift favors approaches that (i) triangulate evidence across perturbation modalities (102), (ii) resolve timing through staged or time-resolved readouts (136), and (iii) increase phenotypic resolution using single-cell and high-content measurements that distinguish direct viral effects from secondary stress responses (38). In parallel, validation is trending toward more physiological systems, including primary cells, organoids, co-cultures, and *in vivo* models, so intervention targets are selected for robustness, not convenience.

These discovery advances sharpen constraints on intervention choice. Many attractive host nodes sit in core cellular processes, so efficacy must be weighed against toxicity, compensatory remodeling, and dysregulation risk. These requirements increasingly shape what good targets look like, often favoring intervention points that are conditionally engaged during infection, cell-type restricted, or transiently modulated rather than ablated. Even state reprogramming approaches must contend with the possibility that engineered antiviral states may inadvertently compromise differentiation, proliferation, or response to other environmental cues, risks requiring rigorous evaluation in relevant physiological models.

Across modalities, delivery and system-level integration remain decisive. Vector or nanoparticle choice matters, but so does host biology: transduction efficiency, tissue tropism, and expression durability can be governed by host gene networks, implying delivery performance is partly predictable and potentially engineerable. This reframes delivery from a purely materials problem into a joint optimization problem spanning payload, vehicle, and host state. Different platforms impose distinct tradeoffs: viral vectors support durable expression but face constraints from immunity, cargo limits, and inter-individual variability (137); lipid nanoparticles enable repeatable transient dosing but often struggle with tissue specificity and heterogeneous biodistribution (138); *ex vivo* engineered cell therapies allow complex circuits and safety features to be assembled before infusion, but are constrained by manufacturing and by engineering cells’ ability to access relevant anatomical niches (139). For many proposed synthetic antivirals, targeting the right cells at the right time will be a prerequisite for durable *in vivo* efficacy.

Looking ahead, progress will likely come from scaling screens while strengthening feedback between discovery and design. Three directions stand out. First, context-aware functional genomics, standardizing cross-model comparisons, expanding physiological systems, and adopting richer phenotypes should yield condition-tagged dependency maps specifying when each mechanism holds. Second, predictive modeling will be increasingly important, integrating perturbational data sets with single-cell atlases and quantitative infection dynamics to generate design rules forecasting efficacy, toxicity, and escape risk before building a system. Third, synthetic biology will continue shifting toward composable, rigorously testable modules, including infection-state sensors, antiviral actuators, and control layers such as logic gating, tunable dosing, and safety switches, enabling therapeutic tuning within a defined window rather than maximizing potency alone.
